# A Mobile Anchor Node Assisted RSSI Localization Scheme in Underwater Wireless Sensor Networks

**DOI:** 10.3390/s19204369

**Published:** 2019-10-10

**Authors:** Yanlong Sun, Yazhou Yuan, Qimin Xu, Changchun Hua, Xinping Guan

**Affiliations:** 1 Institute of Electrical Engineering, Yanshan University, Qinhuangdao 066004, China; yanlongsun@stumail.ysu.edu.cn (Y.S.); cch@ysu.edu.cn (C.H.); 2 Department of Automation, Shanghai Jiao Tong University, Shanghai 200240, China; qiminxu@sjtu.edu.cn (Q.X.); xpguan@sjtu.edu.cn (X.G.)

**Keywords:** underwater wireless sensor networks (UWSNs), localization, mobile anchor node, RSSI

## Abstract

In this paper, a mobile anchor node assisted RSSI localization scheme in underwater wireless sensor networks (UWSNs) is proposed, which aims to improve location accuracy and shorten location time. First, to improve location accuracy, we design a support vector regression (SVR) based interpolation method to estimate the projection of sensor nodes on the linear trajectory of the mobile anchor node. The proposed method increases the accuracy of the nonlinear regression model of noisy measured data and synchronously decreases the estimation error caused by the discreteness of measured data. Second, to shorten location time, we develop a curve matching method to obtain the perpendicular distance from sensor nodes to the linear trajectory of the mobile anchor node. The location of the sensor node can be calculated based on the projection and the perpendicular distance. Compared with existing schemes that require the anchor node to travel at least two trajectories, the proposed scheme only needs one-time trajectory to locate sensor nodes, and the location time is shortened with the reduction in the number of trajectories. Finally, simulation results prove that the proposed scheme can obtain more accurate sensor node location in less time compared with the existing schemes.

## 1. Introduction

With the rapid development of communication and sensor technology, underwater wireless sensor networks (UWSNs) have become a fast-growing field [1]. UWSNs have a wide range of applications, such as pollution monitoring, off-shore oil exploration, and oceanography data collection [2,3,4]. In UWSNs, localization is a fundamental issue, because only the data associated with the location of the sensor node can be meaningfully interpreted by data users [5]. Compared with the terrestrial localization issue, underwater localization has some fundamental differences. First, the Global Positioning System (GPS) technology is unavailable in the underwater environment, because of the strong attenuation of radio waves in water [6]. Thus, the underwater node cannot directly locate itself through the GPS. Second, due to the large propagation delay in underwater acoustic channels, the cost of maintaining clock synchronization is high and underwater nodes are usually clock asynchronous [7]. Third, the transmitting energy consumption of the underwater sensor node is several times or even hundreds of times the receiving energy consumption. To save energy, the underwater sensor node should reduce message transmission during the localization process [8].

The localization method based on Received Signal Strength Indicator (RSSI) does not require clock synchronization, which avoids the high cost of maintaining clock synchronization [9]. Localization methods require anchor nodes to provide reference locations [10]; since the cost of the anchor node is much higher than that of the ordinary sensor node, it is expensive to deploy a large number of static anchor nodes in UWSNs [11]. Hence, using a small number of mobile anchor nodes to assist localization is an economical method [12]. Localization schemes [13,14,15] jointly use the RSSI and one mobile anchor node to reduce location cost. Guo [13] proposed a mobile anchor node-assisted localization scheme called Perpendicular Intersection (PI). By comparing the received RSSI values on a node, this scheme achieved the locating of nodes using the geometric relationship between the node and the trajectory of the mobile beacon. In this scheme, the error of determined projection in the localization process led to the error of the node location. To improve location accuracy, Pratap et al. [14] adopted a piecewise polynomial curve fitting method to reduce the error of determined projection. Zhao et al. [15] applied the polynomial curve fitting method and the derivative method to reduce the error of determined projection. Compared with the scheme in [13], the schemes in [14,15] improve the location accuracy. However, there are two points worthy of further study in [13,14,15]: (1) To improve location accuracy, is there any other better way to reduce the error of determined projection than existing schemes? (2) In existing schemes, the anchor node needs to travel at least two trajectories to locate the sensor node. Can the anchor node only travel one trajectory to shorten the location time?

Based on the above two considerations, in this paper, we propose a mobile anchor node assisted RSSI localization scheme in UWSNs. First, to improve location accuracy, we design a support vector regression (SVR) based interpolation method to estimate the projection of sensor nodes on the linear trajectory of the mobile anchor node. The proposed method increases the accuracy of the nonlinear regression model of noisy measured data and synchronously decreases the estimation error caused by the discreteness of measured data. The SVR based interpolation method achieves an improvement in location accuracy by reducing the error between the determined projection and the actual projection. Second, we develop a curve matching method to obtain the perpendicular distance from sensor nodes to the linear trajectory of the mobile anchor node. The location of the sensor node can be calculated based on the projection and the perpendicular distance. Compared with existing schemes that require the anchor node to travel at least two trajectories, the proposed scheme only needs one trajectory to locate sensor nodes, and the location time is shortened with the reduction in the number of trajectories. Finally, simulations prove that, compared with existing schemes, the proposed scheme can obtain more accurate sensor node location in less time. The main contributions of this paper are twofold:

- To improve location accuracy, we design an SVR based interpolation method to estimate the projection of sensor nodes on the linear trajectory of the mobile anchor node. This method increases the accuracy of the nonlinear regression model of noisy measured data and synchronously decreases the estimation error caused by the discreteness of measured data.
- To shorten location time, we develop a curve matching method to obtain the perpendicular distance from sensor nodes to the linear trajectory of the mobile anchor node. Compared with existing schemes, the proposed scheme only needs one trajectory of the mobile anchor node to locate sensor nodes, which significantly shortens the location time.

The remainder of this paper is organized as follows. Section 2 discusses the related work. In Section 3, we describe the details of the proposed localization scheme. Performance evaluations by simulation are presented in Section 4, followed by the conclusion and future work in Section 5.

## 2. Related Works

### 2.1. Overview of Localization Approaches

Generally speaking, localization approaches can be classified into range-free and range-based [16]. Range-free approaches usually employ the local topology connectivity to estimate the location of the sensor node and obtain a coarser location [17]. The authors of [18] proposed a range-free method, named Area Localization Scheme (ALS). In ALS, anchor nodes broadcast acoustic signals that can be transmitted at varying power levels, underwater sensor nodes passively listen to the signals and record the information, and the sink node can use the information to localize the sensor within a certain area. In contrast, range-based approaches determine the location of sensor nodes using various ranging methods and provide an accurate location [19]. Ranging methods can be classified into four categories [20]: Time of Arrival (TOA) [21,22,23,24], Time Difference of Arrival (TDOA) [25,26,27], Angle of Arrival (AOA) [28,29,30] and Received Signal Strength Indicator (RSSI) [31,32,33]. In underwater environments, distance information obtained by the TOA method is usually used, such as wide coverage positioning system (WPS) [34], GPS-less localization protocol (GPS-less) [35], motion-aware sensor localization (MASL) [36], underwater sparse positioning (USP) [37] and 3D underwater localization (3DUL) [38]. TOA and TDOA methods require highly precise time synchronization of sensor nodes, which is hard to achieve due to the characteristics of acoustic signal when traveling in water [7]. The AOA method requires special devices for directional transmission and reception, which increase the cost of localization [19]. The RSSI method is cost-efficient and convenient because it does not require time synchronization and special devices. Since the RSSI value is vulnerable to environment disturbances, it is difficult to achieve accurate ranging by directly mapping the absolute RSSI value to the physical distance [9].

### 2.2. Localization Approaches Related to the Mobile Anchor Node

Both range-free and range-based localization approaches require anchor nodes, which are equipped with GPS units to provide reference locations [10]. Since the cost of the anchor node is much higher than that of the sensor node, it is expensive to deploy a large number of anchor nodes in UWSNs [11]. Hence, using mobile anchor nodes to assist nearby sensor nodes in localization is an economical and flexible approach [12]. In [39], a Dive and Rise (DNR) positioning method is presented, in which mobile anchor nodes are used to replace static ones. While sinking and rising, DNR anchor nodes broadcast their positions. Unknown nodes are localized by passively listening to DNR anchor nodes’ messages. DNR method reduces communication overhead and energy consumption by passively listening, but localization performance heavily depend on frequency of location updates and the number of anchor nodes.

### 2.3. Localization Approaches Jointly Using the RSSI and the Mobile Anchor Node

Localization schemes [13,14,15] not only jointly use the RSSI and the mobile anchor node to reduce location cost, but also use a method of contrasting RSSI values to avoid the inaccurate ranging caused by directly mapping the RSSI value to the physical distance. In [13], a mobile anchor node moves along a linear trajectory and sends a message at each broadcast point on the trajectory. The sensor node measures the RSSI value of each received message. The broadcast point with the maximum measured RSSI value is approximately determined as the projection of the sensor node on the trajectory. Using two non-parallel trajectories, the location of the sensor node can be determined by calculating the intersection of perpendiculars passing through the determined projection on each trajectory. The location accuracy depends on the error between the determined projection and the actual projection. To improve location accuracy, different schemes are proposed to make the determined projection closer to the actual projection. Pratap et al. [14] tried to find an increasing as well as decreasing trends of measured RSSI values for each trajectory, and then used polynomial curve fitting for each trend, and determined the intersection of the increasing trend polynomial and the decreasing trend polynomial as the projection of the node on the trajectory. Zhao et al. [15] applied the polynomial curve fitting method to measured RSSI values, used the derivative method to calculate the point with the maximum value of the polynomial, and determined the point as the projection of the nodes on the trajectory. Compared with the scheme in [13], the schemes in [14,15] reduce the error between the determined projection and the actual projection, and improve the location accuracy. However, the error of the determined projection of existing schemes can be further reduced, and the location accuracy of the sensor node can be further improved. In addition, existing schemes require at least two trajectories; the number of trajectories can be reduced to one and the location time can be further shortened. In this paper, we mainly research these two points, and the details of our proposed localization scheme are given in Section 3.

## 3. Detailed Description of the Proposed Localization Scheme

The proposed localization scheme consists of four steps: Firstly, the network system architecture and trajectory of the mobile anchor node are designed to obtain a set of measured RSSI values. Secondly, an SVR-based interpolation method is designed to process measured RSSI values to estimate the projection of a sensor node on the trajectory. Thirdly, a curve matching method is developed to process measured RSSI values to obtain the perpendicular distance from a sensor node to the trajectory. Finally, the location of the sensor node is calculated by a geometric method. Table 1 shows the main notations used in this paper.

### 3.1. Network System Architecture, Mobile Anchor Node Trajectory, and Obtaining a Set of Measured RSSI Values

#### 3.1.1. Network System Architecture

**Figure 1:**
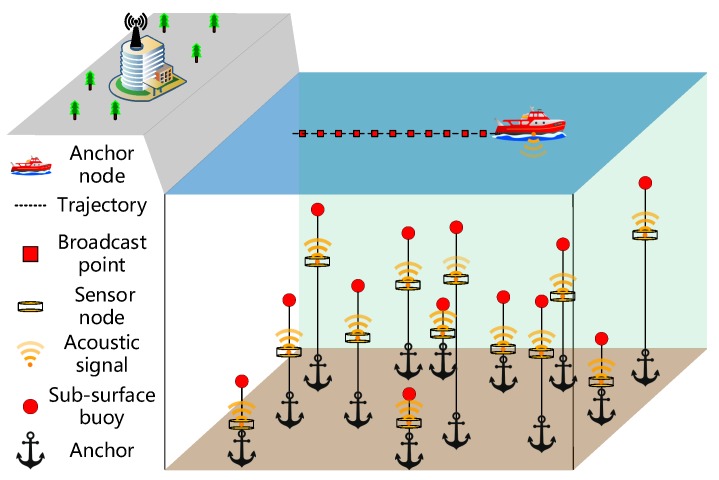
The network system architecture of the proposed localization scheme.

Figure 1 illustrates the network system architecture of the proposed localization scheme, which consists of one mobile anchor node and a number of underwater sensor nodes. The mobile anchor node is a ship which patrols on the sea surface of the sensing area based on a planned trajectory. The mobile anchor node is fitted with a Global Positioning System (GPS) receiver, which can obtain its real-time geographic location. The mobile anchor node can broadcast messages through a spherical omnidirectional acoustic transceiver. Sensor nodes are deployed in the sensing area and stay at their locations by anchor chains and sub-surface buoys. Each sensor node is equipped with an acoustic transceiver, which can receive messages and measure the RSSI value of each message, and has a pressure sensor installed, which can obtain its depth information.

#### 3.1.2. The Trajectory of the Mobile Anchor Node

**Figure 2:**
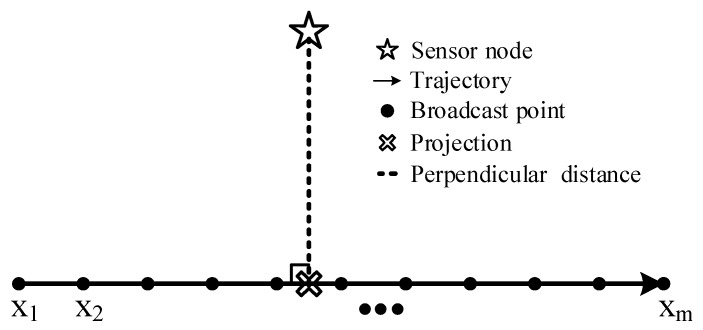
The trajectory of the mobile anchor node.

As shown in Figure 2, the mobile anchor node starts at point $x_{1}$, moves along a linear trajectory, and ends at point $x_{m}$. In the whole process, the mobile anchor node moves at a uniform speed and periodically broadcasts its real-time location message at a fixed transmission power. The length of the trajectory is *L*, the interval between adjacent broadcast points is ΔL, and the number of broadcast points on the trajectory is

(1) m=L/ΔL+1

#### 3.1.3. Obtaining a Set of Measured RSSI Values

When the mobile anchor node moves along the trajectory, the underwater sensor node receives messages and measures the RSSI value of each messages. The measured RSSI value in the underwater environment can be expressed as [40]

(2) $$\tilde{r}\left(l\right)=SL-TL\left(l\right)+\zeta$$

where SL is source level, ζ is a random noise which is assumed to be a zero-mean gaussian random variable with variance $\sigma^{2}$, and TL(l) is the transmission loss for an underwater acoustic signal, which can be expressed as

(3) $$TL\left(l\right)=10\cdot k\cdot \log\left(l\right)+\alpha \cdot l$$

where *k* is the spreading coefficient, α is the absorption coefficient, and *l* is the transmission distance, which is expressed as

(4) $$l=\sqrt{{\left(x-x_{N}\right)}^{2}+{\left(y-y_{N}\right)}^{2}+{\left(z-z_{N}\right)}^{2}}$$

where (x,y,z) is the location of any point on the trajectory and $\left(x_{N},y_{N},z_{N}\right)$ is the location of a sensor node. Since the trajectory is on the surface of the sea and is a straight line, *z* is 0, and *y* can be linearly represented by *x*. We use the *x*-coordinate to indicate the location of broadcast points. The *x*-coordinate of all broadcast points of the trajectory can form an original location vector

(5) $$X=[x_{1},x_{2},\ldots ,x_{m}]$$

All measured RSSI values can also form a vector

(6) $$\tilde{R}=\left[{\tilde{r}}_{1},{\tilde{r}}_{2},\ldots ,{\tilde{r}}_{m}\right]$$

When ζ in Equation (2) is 0, a noiseless RSSI value vector is

(7) $$R=[r_{1},r_{2},\ldots ,r_{m}]$$

The sensor node uses X and $\tilde{R}$ to determine the projection of a sensor node on the trajectory and the perpendicular distance from a node to the trajectory, and then locates itself using the projection and the perpendicular distance.

### 3.2. Determining the Projection of a Node on the Trajectory

**Figure 3:**
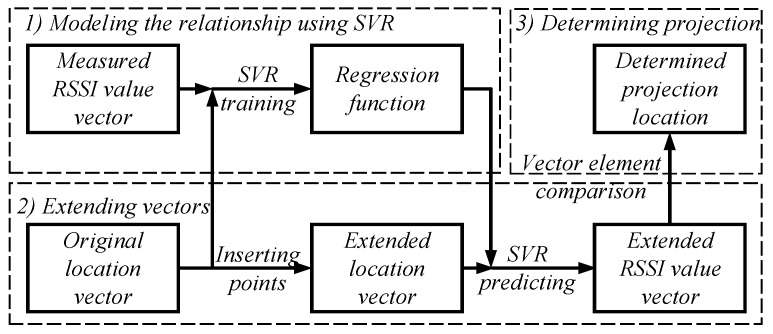
The process of determining the projection of a sensor node on the trajectory.

In this section, to determine the projection of a sensor node on the trajectory, a three-step method is designed, as shown in Figure 3. First, to model the nonlinear relationship between the arbitrary point location and the RSSI value on the trajectory, we perform SVR training on the discrete original location and the measured RSSI value to obtain a regression function. Then, to get more points and RSSI values, we extend the original location vector and the measured RSSI vector. An extended location vector is obtained by uniformly inserting new points in the original location vector, and the extended location vector is input to the regression function to predict the extended RSSI value vector. Finally, to select the point with the largest RSSI value, we compare the elements in the extended RSSI value vector. The point corresponding to the largest RSSI value is determined as the projection of the node on the trajectory.

#### 3.2.1. Modeling the Nonlinear Relationship Using SVR

According to Equations (2)–(4), there is a nonlinear relationship between X and $\tilde{R}$. Since SVR has a rather strong nonlinear approximation ability and antinoise ability [41], we use SVR to learn the regression function by identifying structures in the mapping of input X to output $\tilde{R}$.

The mobile anchor node starts at point $x_{1}$, moves along a linear trajectory, and ends at point $x_{m}$. Then, the node to be located obtains data: the location of *m* broadcast points $x_{1},x_{2},\ldots ,x_{m}$, and the measured RSSI value ${\tilde{r}}_{1},{\tilde{r}}_{2},\ldots ,{\tilde{r}}_{m}$. The location of *i*th broadcast points and the *i*th measured RSSI value together form a data sample $\left\{\left(x_{i},{\tilde{r}}_{i}\right)|x_{i}\in X,{\tilde{r}}_{i}\in \tilde{R},i=1,2,...,m\right\}$. These *m* data samples are used as a dataset for SVR training. We need find an approximation function with the form

(8) $$f\left(x\right)=w\cdot \varphi \left(x\right)+b$$

where $\varphi \left(x\right)$ is a mapping function, *w* is the weight, and *b* is the threshold. Define the ε-insensitive loss function as

(9) $${\left|{\tilde{r}}_{i}-f\left(x_{i}\right)\right|}_{\varepsilon }=\left\{\begin{array}{cc} 0, & \left|{\tilde{r}}_{i}-f\left(x_{i}\right)\right|\leq \varepsilon \\ \left|{\tilde{r}}_{i}-f\left(x_{i}\right)\right|-\varepsilon , & \left|{\tilde{r}}_{i}-f\left(x_{i}\right)\right|>\varepsilon \end{array}\right.$$

By introducing slack variables $\xi_{i}$ and $\xi_{i}^{*}$, the SVR problem is transformed into

(10) $$\min_{w,b,\xi_{i},\xi_{i}^{*}}\frac{1}{2}{∥w∥}^{2}+C\sum_{i=1}^{m}\left(\xi_{i}+\xi_{i}^{*}\right)$$

subject to

(11) $$\left\{\begin{array}{c} f\left(x_{i}\right)-{\tilde{r}}_{i}\leq \varepsilon +\xi_{i}\\ {\tilde{r}}_{i}-f\left(x_{i}\right)\leq \varepsilon +\xi_{i}^{*}\\ \xi_{i},\xi_{i}^{*}\geq 0\\ i=1,2,...,m \end{array}\right.$$

where *C* is the penalty factor. By introducing Lagrange multipliers $\beta_{i},\beta_{i}^{*},\alpha_{i},\alpha_{i}^{*}\geq 0$, the Lagrangian function of Equation (10) can be expressed as

(12) $$\begin{array}{cc} L= & \frac{1}{2}{∥w∥}^{2}+C\sum_{i=1}^{m}\left(\xi_{i}+\xi_{i}^{*}\right)-\sum_{i=1}^{m}\beta_{i}\xi_{i}-\sum_{i=1}^{m}\beta_{i}^{*}\xi_{i}^{*}\\ \; & +\sum_{i=1}^{m}\alpha_{i}\left(f\left(x_{i}\right)-{\tilde{r}}_{i}-\varepsilon -\xi_{i}\right)\\ \; & +\sum_{i=1}^{m}\alpha_{i}^{*}\left({\tilde{r}}_{i}-f\left(x_{i}\right)-\varepsilon -\xi_{i}^{*}\right) \end{array}$$

Letting the partial derivative of a function L with respect to the variable $w,b,\xi_{i},\xi_{i}^{*}$ be equal to 0, we can obtain

(13) $$\begin{array}{cc} w= & \sum_{i=1}^{m}\alpha_{i}^{*}x_{i}-\sum_{i=1}^{m}\alpha_{i}x_{i} \end{array}$$

(14) $$\begin{array}{cc} 0= & \sum_{i=1}^{m}\left(\alpha_{i}^{*}-\alpha_{i}\right) \end{array}$$

(15) $$\begin{array}{cc} C= & \alpha_{i}+\beta_{i} \end{array}$$

(16) $$\begin{array}{cc} C= & \alpha_{i}^{*}+\beta_{i}^{*} \end{array}$$

Substituting Equations (13)–(16) into Equation (12), we can obtain

(17) $$\begin{array}{c} \max_{\alpha ,\alpha^{*}}(-\frac{1}{2}\sum_{i=1}^{m}\sum_{j=1}^{m}\left(\alpha_{i}^{*}-\alpha_{i}\right)\left(\alpha_{j}^{*}-\alpha_{j}\right)K\left(x_{i},x_{j}\right)\\ -\varepsilon \sum_{i=1}^{m}\left(\alpha_{i}^{*}+\alpha_{i}\right)+\sum_{i=1}^{m}{\tilde{r}}_{i}\left(\alpha_{i}^{*}-\alpha_{i}\right)) \end{array}$$

subject to

(18) $$\left\{\begin{array}{c} \sum_{i=1}^{m}\left(\alpha_{i}^{*}-\alpha_{i}\right)=0\\ 0\leq \alpha_{i},\alpha_{i}^{*}\leq C \end{array}\right.$$

where $K\left(x_{i},x_{j}\right)$ is a RBF kernel function

(19) $$K\left(x_{i},x_{j}\right)=\exp\left(-\frac{{∥x_{i}-x_{j}∥}^{2}}{\sigma^{2}}\right)$$

KKT conditions for Equation (18) are

(20) $$\left\{\begin{array}{c} \alpha_{i}\left(f\left(x_{i}\right)-{\tilde{r}}_{i}-\varepsilon -\xi_{i}\right)=0\\ \alpha_{i}^{*}\left({\tilde{r}}_{i}-f\left(x_{i}\right)-\varepsilon -\xi_{i}^{*}\right)=0\\ \beta_{i}\xi_{i}=0,\beta_{i}^{*}\xi_{i}^{*}=0\\ \left(C-\alpha_{i}\right)\xi_{i}=0,\left(C-\alpha_{i}^{*}\right)\xi_{i}^{*}=0 \end{array}\right.$$

The final regression function is obtained:

(21) $$f\left(x\right)=\sum_{i=1}^{m}\left(\alpha_{i}^{*}-\alpha_{i}\right)K\left(x,x_{i}\right)+b$$

The regression function is the nonlinear relationship between X and $\tilde{R}$. We use the regression function to predict the location vector X, and obtain the predicted RSSI vector

(22) $$\hat{R}=\left[{\hat{r}}_{1},{\hat{r}}_{2},\ldots ,{\hat{r}}_{m}\right]$$

**Figure 4:**
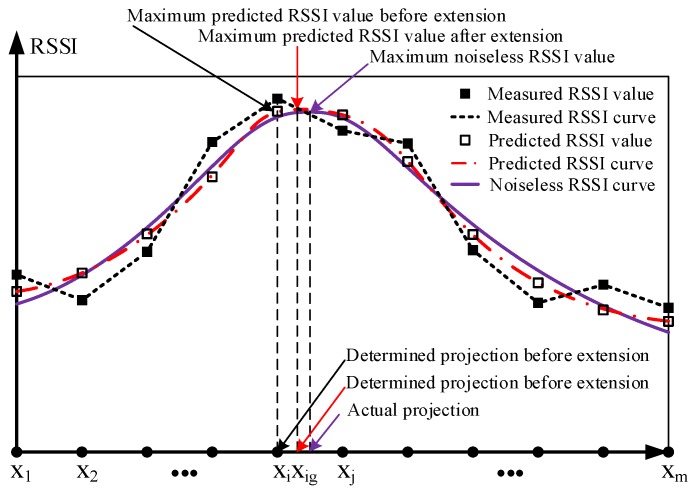
Determining the projection of a node on the trajectory by the SVR-based interpolation method.

We embed X of Equation (5), $\tilde{R}$ of Equation (6), R of Equation (7), and $\hat{R}$ of Equation (22) into Figure 4. The measured RSSI curve (black dashed line) is drawn using all the elements in $\tilde{R}$. The predicted RSSI curve (red dash-dot line) is drawn using all the elements in $\hat{R}$. The noiseless RSSI curve (purple solid line) is drawn using all the elements in R. Since the SVR has a good noise reduction effect, the predicted RSSI curve should be closer to the noiseless RSSI curve than the measured RSSI curve.

#### 3.2.2. Extending Vectors

Since the trained regression function by SVR is a black box, it can transform the input into the output, but the inner working is difficult to interpret because the underlying models are based on complex mathematical systems [42]. The derivative method cannot be used to find the point with the maximum value on the predicted RSSI curve. We extend the location vector X and predicted RSSI vector $\hat{R}$ to determine a point closer to the actual projection.

**Figure 5:**
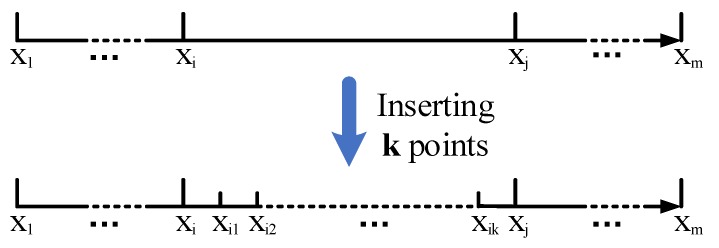
The extension of the location vector.

As shown in Figure 4, the predicted RSSI curve has first and second maximum RSSI values at points $x_{i}$ and $x_{j}$, respectively. Before extending vectors, $x_{i}$ is determined as the projection, but has a large error with the actual projection. Although shortening the distance between broadcast points can reduce the error, it is difficult to reduce this distance to a small value in an actual sea environment. To determine a point closer to the actual projection position, we evenly insert *k* points between $x_{i}$ and $x_{j}$, as shown in Figure 5. The extended location vector is

(23) $$\overset{`}{X}=\left[{\overset{`}{x}}_{1},\ldots ,{\overset{`}{x}}_{i},{\overset{`}{x}}_{i1},{\overset{`}{x}}_{i2},\ldots ,{\overset{`}{x}}_{ik},{\overset{`}{x}}_{j},\ldots ,{\overset{`}{x}}_{m}\right]$$

After using the regression function to predict all the elements in $\overset{`}{X}$, the extended RSSI vector can be obtained

(24) $$\overset{`}{R}=\left[{\overset{`}{r}}_{1},\ldots ,{\overset{`}{r}}_{i},{\overset{`}{r}}_{i1},{\overset{`}{r}}_{i2},\ldots ,{\overset{`}{r}}_{ik},{\overset{`}{r}}_{j},\ldots ,{\overset{`}{r}}_{m}\right]$$

#### 3.2.3. Determining the Projection

We find the maximum element in $\overset{`}{R}$ by the element comparison, and determine the corresponding element in $\overset{`}{X}$ as the projection of a node on the trajectory. As shown in Figure 4, the predicted RSSI curve has the maximum predicted RSSI value at $x_{ig}$ after vectors are extended, and $x_{ig}$ is determined as the projection of the node. $x_{ig}$ is closer to the actual projection than $x_{i}$, and the error between the determined projection and the actual projection position is reduced.

### 3.3. Determining the Perpendicular Distance from a Node to the Trajectory

**Figure 6:**
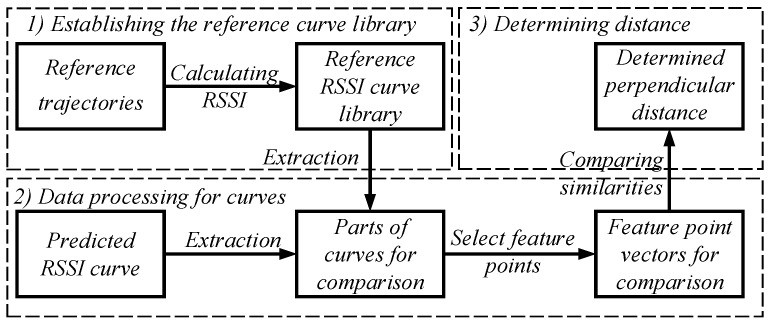
The process of determining the perpendicular distance from a node to the trajectory.

In this section, to determine the perpendicular distance from a node to the trajectory, we design a three-step method, as shown in Figure 6. First, to establish the reference RSSI curve library, we calculate the RSSI value of the broadcast point on n reference trajectories. Then, to accurately compare the similarities between curves, we extract the parts to be compared from the predicted curve and reference curves, and select the feature points on the extracted parts, so as to get the feature point vectors for comparison. Finally, to select the feature point vector with the highest similarity, we compare the similarity between the feature point vectors. The distance corresponding to the selected feature point vector is determined as the perpendicular distance from the node to the trajectory.

#### 3.3.1. Establishing the Reference RSSI Curve Library

**Figure 7:**
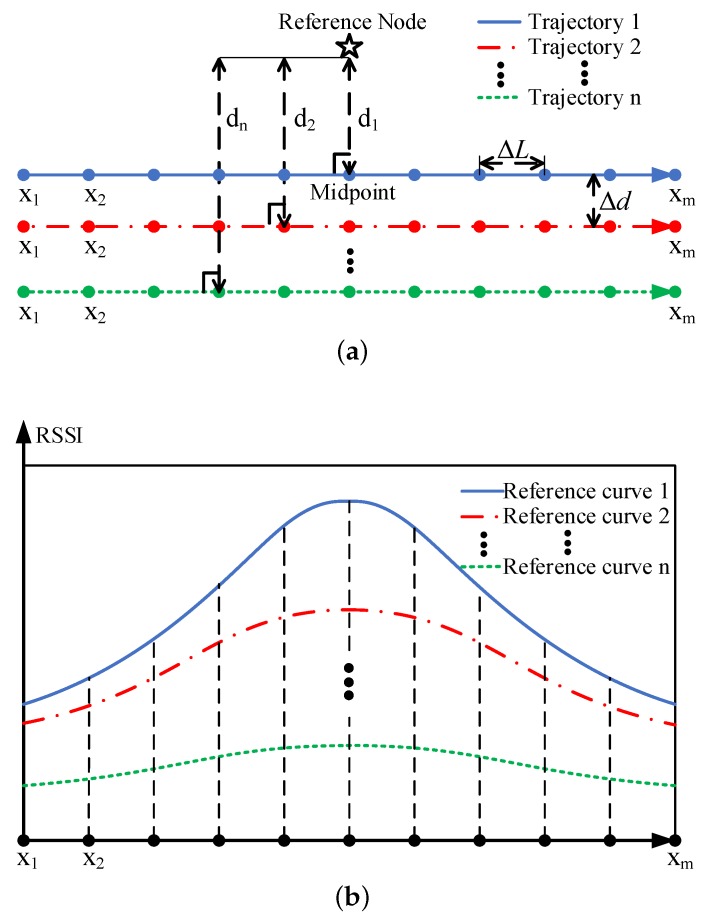
The establishment of the reference RSSI curve library: (**a**) the positional relationship between the reference node and *n* trajectories; and (**b**) the reference RSSI curve library.

As shown in Figure 7a, there are *n* parallel and identical trajectories with *m* broadcast points on each trajectory. The spacing between adjacent trajectories is Δd, and the interval between adjacent broadcast points is ΔL. The projection of the reference node on all the trajectories are their respective midpoints. The perpendicular distance from the reference node to trajectory 1, trajectory 2, ..., trajectory n are $d_{1}$, $d_{2}$, ..., $d_{n}$, respectively. When the random noise ζ in Equation (2) is 0, we name the RSSI value received by the reference node as the reference RSSI value. Each trajectory corresponds to a reference RSSI curve, as shown in Figure 7b. For the *i*th trajectory, a reference RSSI vector $R_{i}$ can be constructed using the reference RSSI value of the broadcast point at $x_{1}$, $x_{2}$, ..., $x_{m}$,

(25) $$R_{i}=\left[r_{i1},r_{i2},\ldots ,r_{im}\right]$$

We use $R_{i}$ to represent the *i*th reference RSSI curve. All reference RSSI curves can establishing a reference RSSI curve library, which can be expressed as

(26) $$L_{c}=\left\{\begin{array}{c} R_{1}=\left[r_{11},r_{12},\ldots ,r_{1m}\right]\\ R_{2}=\left[r_{21},r_{22},\ldots ,r_{2m}\right]\\ \vdots \\ R_{n}=\left[r_{n1},r_{n2},\ldots ,r_{nm}\right] \end{array}\right.$$

Since the perpendicular distance from the node to each trajectory is different, the transmission distance from the same broadcast point of each trajectory to the reference node are different, the reference RSSI values of the respective trajectories at the same broadcast point are different, and the reference RSSI vector of each trajectory are different. Therefore, there is a mapping relationship between the perpendicular distance from the reference node to the trajectory and the reference RSSI curve, which can be expressed as

(27) $$d_{i}=F\left(R_{i}\right)\;$$

where i=1,2,...,n, *F* represents the mapping relationship. All mapping relationships between the reference RSSI vector and its perpendicular distance can establish a mapping relationship library, which can be expressed as

(28) $$L_{r}=\left\{\begin{array}{c} d_{1}=F\left(R_{1}\right)\\ d_{2}=F\left(R_{2}\right)\\ \vdots \\ d_{n}=F\left(R_{n}\right) \end{array}\right.$$

#### 3.3.2. Data Processing for Curves Used for Comparison

Three steps of data processing are required before the measured RSSI curve is compared with each curve in the reference RSSI curve library. Firstly, the measured RSSI value contains noise which would cause a large error in the result of the curve matching. Since SVR has a strong ability [41], we use the predicted RSSI curve instead of the measured RSSI curve to perform curve matching.

Secondly, the projection of a node on the trajectory is difficult to coincide with the midpoint of the trajectory, which causes the predicted RSSI curve to be offset from the reference RSSI curve. This offset causes us to extract only a part of the predicted RSSI curve to compare with the corresponding part of the reference RSSI curve.

**Figure 8:**
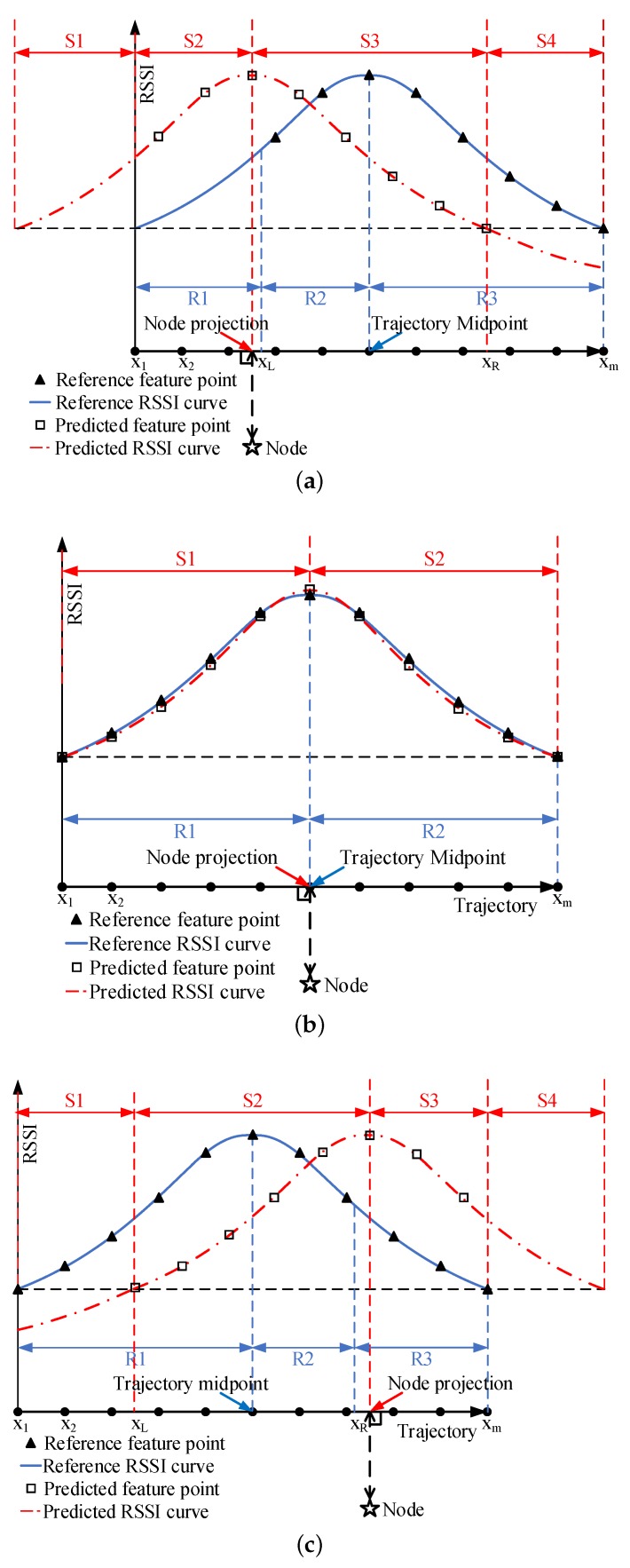
Three positional relationships between the projection of the node and the midpoint of the trajectory: (**a**) the projection of the node is on the left of the midpoint; (**b**) the node projection coincides with the midpoint; and (**c**) the projection of the node is on the right of the midpoint.

As shown in Figure 8, the projection of the node has three positional relationships with the midpoint of the trajectory: on the left (Figure 8a), coincident (Figure 8b), and on the right (Figure 8c). When the projection of the node is on the left of the midpoint, the predicted RSSI curve has a left offset relative to the reference RSSI curve. The predicted RSSI curve can be divided into four parts: S1, S2, S3, and S4. The reference RSSI curve can be divided into three parts: R1, R2, and R3. The S1 part of the predicted RSSI curve corresponds to the R1 part of the reference RSSI curve. However, the left offset of the curve causes the trajectory of the S1 part to exceed the actual travel range of the mobile anchor node, that is, the S1 part does not exist, and does not need to be compared with R1. The trajectory of the S4 part is within the range of actual travel of the mobile anchor node. However, the distance from the node to the point on the S4 part of the trajectory is too large, so that there is no part on the reference RSSI curve that can be compared with S4. Therefore, when the projection of the node is on the left of the midpoint, S2 + S3 of the predicted RSSI curve are compared with R2 + R3 of the reference RSSI curve. Similarly, when the node projection coincides with the midpoint, S1 + S2 of the predicted RSSI curve are compared with R1 + R2 of the reference RSSI curve. When the projection of the node is on the right of the midpoint, S2 + S3 of the predicted RSSI curve are compared with R1 + R2 of the reference RSSI curve.

Thirdly, we need select feature points on the predicted RSSI curve to compare with the corresponding feature points on the reference RSSI curve. As shown in Figure 8a, when the projection of the node is on the left of the midpoint, assuming that the projection of the node on the trajectory is $x_{pro}$, the midpoint of the trajectory is $x_{mid}$. The offset of the predicted RSSI curve from the reference RSSI curve is

(29) $$L_{off}=\left|x_{mid}-x_{pro}\right|$$

Starting from the projection, we select a feature point every ΔL in the S2 part and the S3 part, respectively. The number of feature points in the S2 part, the S3 part, the S2 + S3 part are NL, NR, and NT, respectively. They can be expressed as

(30) $$\left\{\begin{array}{cc} NL & =\left\lfloor (L/2-L_{off})/\Delta L\right\rfloor \\ NR & =\left\lfloor (L/2)/\Delta L\right\rfloor \\ NT & =NL+1+NR \end{array}\right.$$

The location of the leftmost feature point is

(31) $${\overset{ˇ}{x}}_{1}=x_{pro}-NL*\Delta L$$

and the location of any feature point in S2 + S3 is

(32) $${\overset{ˇ}{x}}_{i}={\overset{ˇ}{x}}_{1}+\left(i-1\right)*NT*\Delta L$$

where i=1,2...,NT.

The vector formed by the location of each feature point is

(33) $$\begin{array}{c} \overset{ˇ}{X}=\left[{\overset{ˇ}{x}}_{1},{\overset{ˇ}{x}}_{2},\ldots ,{\overset{ˇ}{x}}_{NT}\right] \end{array}$$

We use the obtained regression function in Section 3.2 to predict each element in $\overset{ˇ}{X}$, and obtain the RSSI value vector of the feature point, which can be expressed as

(34) $$\begin{array}{c} \overset{ˇ}{R}=\left[{\overset{ˇ}{r}}_{1},{\overset{ˇ}{r}}_{2},\ldots ,{\overset{ˇ}{r}}_{NT}\right] \end{array}$$

We select the broadcast point of the R2 + R3 of the reference RSSI curve as the location of the feature point, and the RSSI value corresponding to the broadcast point as the value of the feature point. The location vector $\bar{X}$ and RSSI value vector $\bar{R}$ formed by the feature points on the R2 + R3 of the *i*th reference RSSI curve are

(35) $$\left\{\begin{array}{cc} \bar{X} & =[x_{m-(N_{t}-1)},x_{m-(N_{t}-2)},\ldots ,x_{m}]\\ {\bar{R}}_{i} & =[r_{i(m-\left(N_{t}-1\right))},r_{i(m-\left(N_{t}-2\right))},\ldots ,r_{im}] \end{array}\right.$$

$\overset{ˇ}{R}$ and ${\bar{R}}_{i}$ are used for similarity comparison. When the node projection coincides with the midpoint, $\overset{ˇ}{R}$ and ${\bar{R}}_{i}$ are

(36) $$\left\{\begin{array}{cc} \overset{ˇ}{R} & =[{\hat{r}}_{1},{\hat{r}}_{2},\ldots ,{\hat{r}}_{m}]\\ {\bar{R}}_{i} & =[r_{i1},r_{i2},\ldots ,r_{im}] \end{array}\right.$$

When the projection of the node is to the right of the midpoint, some equations that differ from the projection on the left of the midpoint are

(37) $$\begin{array}{cc} NL & =\left\lfloor (L/2)/\Delta L\right\rfloor \end{array}$$

(38) $$\begin{array}{cc} NR & =\left\lfloor (L/2-L_{off})/\Delta L\right\rfloor \end{array}$$

(39) $$\begin{array}{cc} {\bar{R}}_{i} & =[r_{i1},r_{i2},\ldots ,r_{i(m-(NT-1))}] \end{array}$$

#### 3.3.3. Determining the Perpendicular Distance by Comparing Curve Similarities

We need compare the similarity between $\overset{ˇ}{R}$ and each ${\bar{R}}_{i}$, find the ${\bar{R}}_{i}$ that is most similar to $\overset{ˇ}{R}$, and determine the $d_{i}$ corresponding to the ${\bar{R}}_{i}$ as the perpendicular distance from the node to the trajectory. Before calculating the similarity, it is necessary to normalize the elements of the vector to avoid the weight of the elements with larger absolute values in the similar calculation results being too large. We combine the $\overset{ˇ}{R}$ with all ${\bar{R}}_{i}$ to construct a new matrix

(40) $$\begin{array}{c} A={\left[\overset{ˇ}{R},{\bar{R}}_{1},{\bar{R}}_{2},\ldots ,{\bar{R}}_{n}\right]}^{T} \end{array}$$

*x* is the element at the *i*th row and *j*th column of the matrix A, and the normalized value of *x* is

(41) $$y=\left(x-x_{mean}\right)\times \left(y_{std}/x_{std}\right)+y_{mean}$$

where $x_{mean}$ is the mean value for the *j*th column of the matrix A, $x_{std}$ is the standard deviation for the *j*th column of the matrix A, $y_{mean}$ is 0, and $y_{std}$ is 1. The normalized vector $\overset{ˇ}{R}$ is represented as ${\overset{ˇ}{R}}^{{}^{\prime }}$, the normalized vector ${\bar{R}}_{i}$ is represented as ${\bar{R}}_{i}^{{}^{\prime }}$, We compare the similarity of ${\overset{ˇ}{R}}^{{}^{\prime }}$ and ${\bar{R}}_{i}^{{}^{\prime }}$ by calculating the Euclidean distance:

(42) $$E_{i}=∥{\overset{ˇ}{R}}^{{}^{\prime }}-{\bar{R}}_{i}^{{}^{\prime }}∥$$

The smaller is the value of $E_{i}$, the higher is the similarity between vectors. The reference RSSI curve with the smallest euclidean distance is most similar to the predicted RSSI curve. According to Equation (27), the perpendicular distance corresponding to the reference curve is determined as the perpendicular distance from the node to the trajectory.

### 3.4. Calculating the Node Location

In this section, the location of the sensor node is determined using the obtained projection in Section 3.2 and perpendicular distance in Section 3.3. We first transform the three-dimensional (3D) underwater sensor node localization problem into a two-dimensional (2D) localization problem. Then, we describe in detail the calculation process of localization.

#### 3.4.1. Transforming 3D Localization into 2D Localization

**Figure 9:**
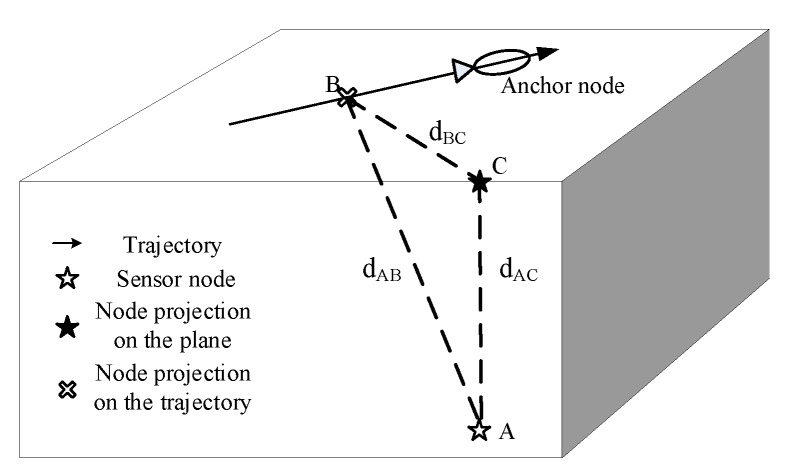
Transforming 3D localization into 2D localization.

As shown in Figure 9, the to-be-localized node A is at the lower right of the trajectory, B is the projection of node A on the trajectory, and C is the projection of node A on the trajectory plane. The coordinates of point A, B, and C are $\left(x_{A},y_{A},z_{A}\right)$, $\left(x_{B},y_{B},0\right)$, and $\left(x_{C},y_{C},0\right)$, respectively. $\left(x_{B},y_{B},0\right)$ can be obtained by the method in Section 3.2. $d_{AB}$ is the perpendicular distance from node A to the trajectory, which can be obtained by the method in Section 3.3. $d_{AC}$ or $z_{A}$ is the depth of node A, which can be measured by a pressure sensor. $d_{BC}$ is the projection of $d_{AB}$ on the plane, as well as the perpendicular distance from C to the trajectory. ΔABC is a right-angle triangle, and $d_{BC}$ can be calculated as

(43) $$d_{BC}=\sqrt{d_{AB}^{2}-d_{AC}^{2}}$$

After the above operations, the task of localizing node A in a 3D space has been transformed into localizing C in a 2D plane.

#### 3.4.2. The Calculation Process of Localization

The equation of the trajectory can be expressed as

(44) y=k×x+b

*k* and *b* can be obtained by bringing the coordinates of two broadcast points into Equation (44). The coordinates of point *C* can be computed by solving

(45) $$\begin{array}{c} \left\{\begin{array}{cc} \; & d_{BC}=\frac{|k\times x_{C}-y_{C}+b|}{\sqrt{k^{2}+1}}\\ \; & \frac{y_{C}-y_{B}}{x_{C}-x_{B}}=-\frac{1}{k} \end{array}\right. \end{array}$$

**Figure 10:**
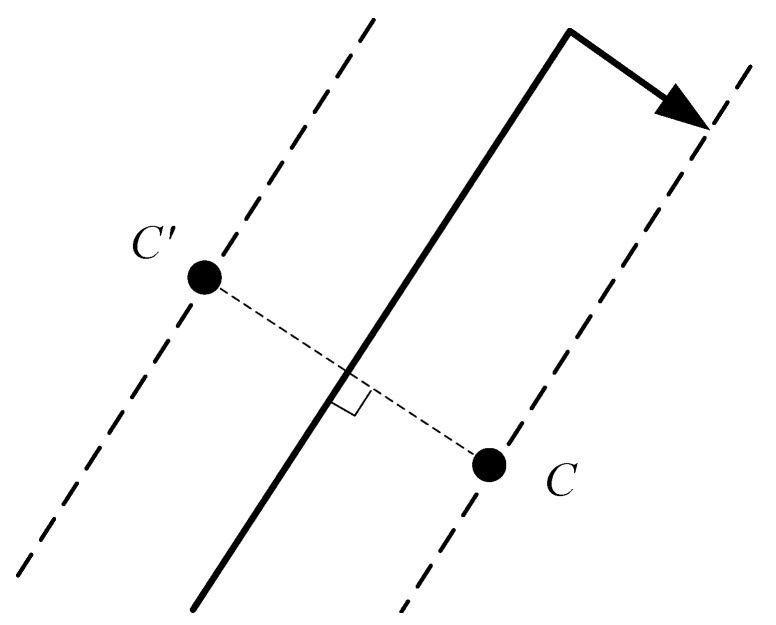
Judging the location of the point C.

However, solving Equation (45) will get two candidate positions, as shown in Figure 10. To determine the true position of point C, when the anchor node reaches the end of the trajectory, it continues to travel a distance along the right perpendicular line of the trajectory. If RSSI values received by the node become larger relative to the RSSI value of the end point, point C in Figure 10 is the true position. If RSSI values received by the node become smaller relative to the RSSI value of the end point, then point $C^{{}^{\prime }}$ in Figure 10 is the true position. After obtaining the true coordinates of point C, the coordinates of point A can be determined as $\left(x_{C},y_{C},z_{A}\right)$.

## 4. Simulation

The performance of the proposed schemes was evaluated through MATLAB simulations. Since the proposed scheme addresses the shortcomings of the PI scheme and the DuRT scheme, we compared our scheme with the PI scheme [13] and the DuRT scheme [14] in the simulation. In [14], the DuRT scheme actually includes three localization methods, DuRT-M1, DuRT-M2, and DuRT-ALL, and the working conditions of the three methods are different. In our simulation, the conditions for using DuRT-M1 were satisfied, but the conditions for using DuRT-M2 and DuRT-ALL were not satisfied. Thus, in the simulation, we used DuRT-M1. For convenience, DuRT-M1 is denoted as DuRT.

### 4.1. Simulation Parameter Setting

Since the PI scheme and DuRT scheme require two trajectories, we designed a triangular prism sensing area. The top of the triangular prism is an equilateral triangle, the left side of the top triangle is the trajectory 1 of the mobile anchor node, and the right side of the top triangle is the trajectory 2. The length of the trajectory is L=600 m, and the depth of the triangular prism is H=100 m. N=100 nodes are randomly deployed in a triangular prism. The interval between adjacent broadcast points ΔL was set to 1 m, 5 m, 10 m, 15 m, and 20 m. The interval between adjacent extended broadcast points is $\Delta L_{ext}=0.5$ m. The spacing between adjacent reference trajectories Δd is 1 m. The acoustic parameters are: SL=100 db, f=24 KHz, and k=2. The speed of the ship is v=5 m/s. In the localization process of this paper, the underwater sensor node does not need to transmit messages, thus the coverage distance of the underwater sensor node is short and is set to $cd_{s}=100$ m. The mobile anchor node needs a large enough coverage distance so that the transmitted message can be received by all sensor nodes in the area, and is set to $cd_{a}=600$ m. For the convenience of viewing, the parameter settings of the simulation are as shown in Table 2.

### 4.2. Simulation Results and Discussion

In this section, we provide the system architecture used in the simulation, and analyze simulation results from three aspects. First, we use three schemes to determine the projection of the node, and compare the mean error of node projections. Then, we compare the measured RSSI curve with the selected RSSI curve, and analyze the error of the perpendicular distance. Finally, we compare the mean location error of the three schemes.

#### 4.2.1. The Network System Architecture in the Simulation

**Figure 11:**
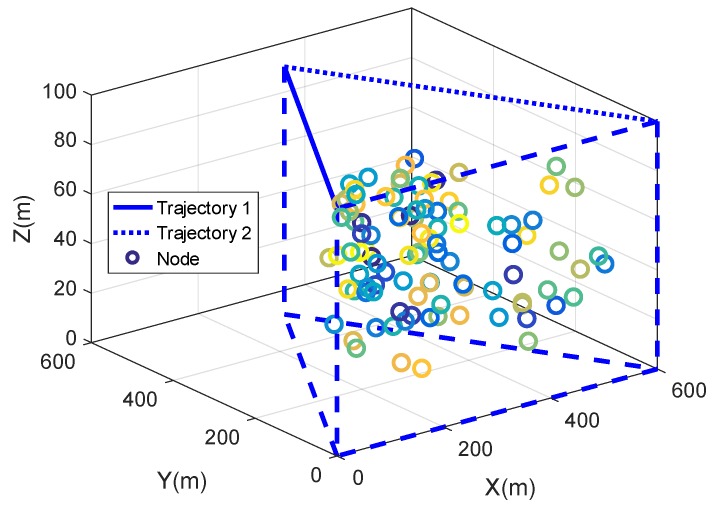
The network system architecture in the simulation.

As shown in Figure 11, 100 nodes (circles of various colors) are randomly deployed in the triangular prism area. The mobile anchor node starts at (0,0,100), moves along trajectory 1 (blue solid line) and trajectory 2 (blue dotted line), and ends at (600,0,100). After the mobile anchor node finishes moving, underwater sensors use receiving messages to locate themselves. The localization process needs to run five times. In the five localization processes, the interval between adjacent broadcast points ΔL is set to 1 m, 5 m, 10 m, 15 m, and 20 m, respectively.

#### 4.2.2. The Error of a Node Projection on the Trajectory

**Figure 12:**
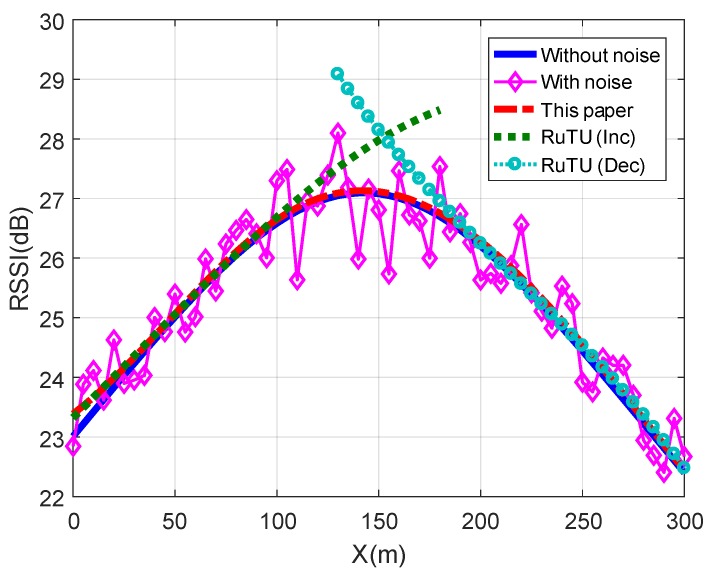
Determining the projection of one node on the trajectory by three schemes (ΔL=10 m).

The PI scheme, the RuDT scheme and the scheme proposed in this paper must first determine the projection position of the node on the trajectory. Therefore, we first compare the error of one node projection of different schemes. As shown in Figure 12, the actual projected position of the node on the trajectory is the position corresponding to the highest point of the curve without noise (Blue curve). The PI scheme selects the position with the largest value on the RSSI curve with noise (Magenta curve with diamond) as the node projection. The DuRT scheme uses a polynomial fitting method to fit an increased curve (Green dotted line) to the left part and a decreased curve (Light blue dotted line with circles) to the right part. The position corresponding to the intersection of the increased curve and the decreased curve is the node projection determined by the DuRT scheme. The scheme in this paper uses SVR to fit a curve and select the position corresponding to the maximum value of this curve as the node projection. The actual projection position is 141. The projection positions determined by the PI scheme, the DURT scheme, and the scheme in this paper are 130, 155, and 140, respectively. The scheme in this paper has the least error of the three schemes.

**Figure 13:**
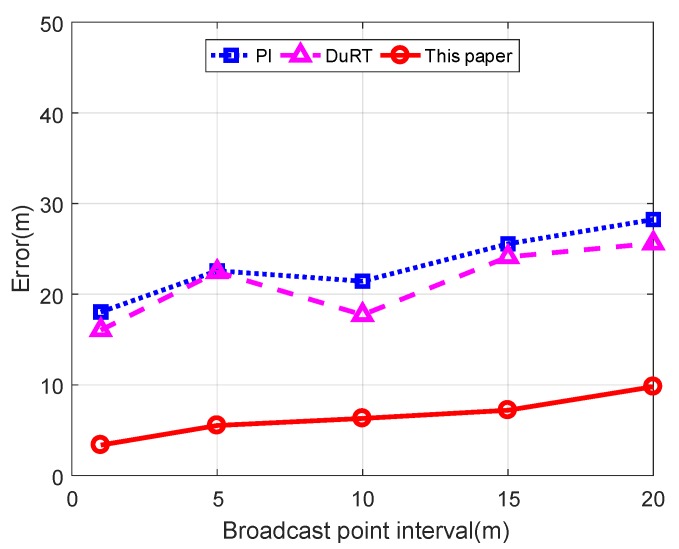
Mean error of the projection of the node on the trajectory determined by different schemes.

As shown in Figure 13, we compare the mean error of node projections for 100 nodes at different broadcast point intervals in three schemes. The mean error of the three schemes increases as the broadcast point interval increases. In the case of the same broadcast point interval, the mean error of the three schemes is sorted from biggest to smallest: the PI scheme, the RuDT scheme, and the scheme proposed in this paper. Due to the influence of noise, the broadcast position with the largest RSSI value is often not the closest broadcast position to the actual projection, as shown in Figure 13. Since the PI scheme only selects the broadcast position with the largest RSSI value, the PI scheme has a large error in determining the node projection. Although the polynomial curve fitting method can fit all RSSI values, the fitting effect is poor. Thus, the DuRT scheme fits the RSSI values on the left and right sides separately. When the left polynomial and the right polynomial have an intersection between the broadcast position with the largest RSSI value and the broadcast position with the second largest RSSI value, the DuRT scheme selects this intersection as the node projection. However, this intersection can only be controlled between two broadcast locations, and it is not possible to control the intersection to be close to the actual projection. Thus, the error of the DuRT scheme is smaller than the error of the PI method, but it is still very large. The scheme in this paper solves the noise problem and fits a good curve to all RSSI values. Therefore, our scheme is an effective scheme for reducing the error of the determined node projection.

#### 4.2.3. The Error of the Perpendicular Distance from the Node to the Trajectory

**Figure 14:**
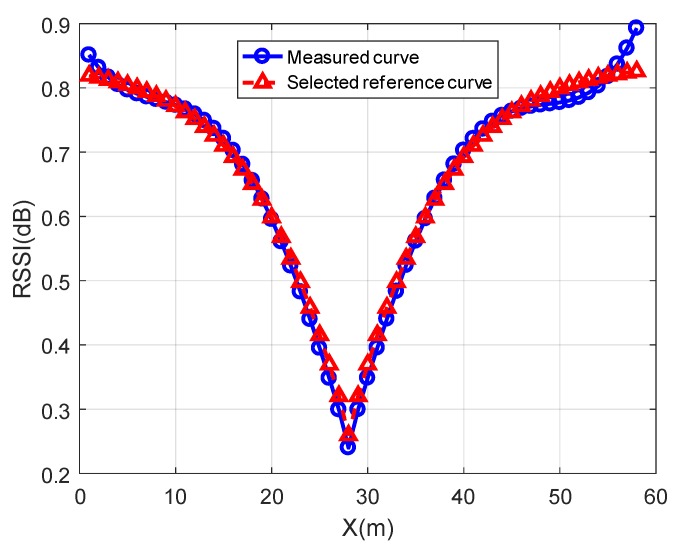
Comparison of curves after data processing.

In Figure 14, the blue curve with circles is the measured curve after data processing, and the red curve with triangles is the selected reference curve after data processing. After the normalization operation, the range of the RSSI curve is reduced, which narrows the weight difference between the comparison results of each feature point in all comparison results. At the same time, the shape of the two curves has been greatly changed from that of Figure 12 after normalization. The total length of the X-axis of the two curves is shortened from 300 to 60, indicating that only a part of the curve is compared. The marks on the two curves are the selected feature points. As shown in Figure 14, the measured curve is very similar to the selected reference curve, so the perpendicular distance corresponding to the reference curve can be determined as the perpendicular distance from the node to the trajectory.

**Figure 15:**
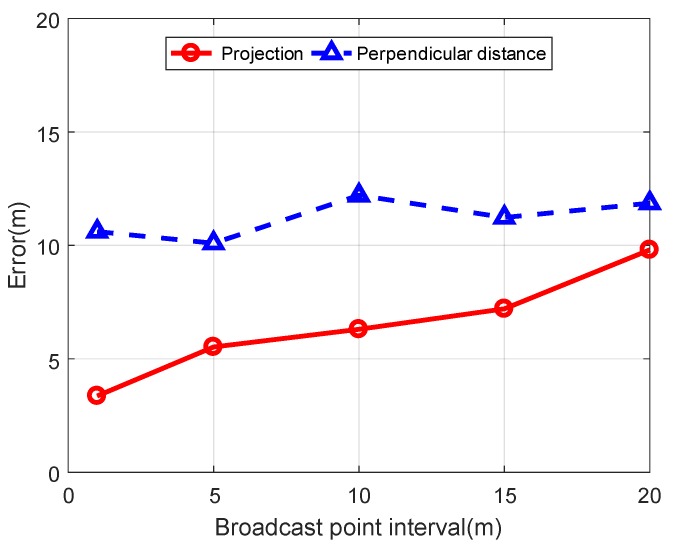
Comparison of mean error between node projection and Perpendicular distance.

As shown in Figure 15, the mean error of the perpendicular distance does not become larger as the broadcast point interval becomes larger, and it is greater than the mean error of the projection.

#### 4.2.4. The Error of the Node Location

**Figure 16:**
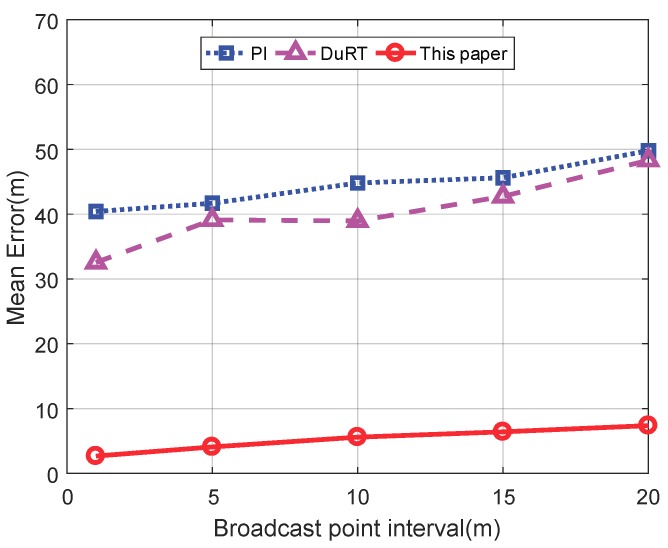
Comparison of the mean location error of the three schemes.

Figure 16 shows the comparison of the mean location error of the three schemes. In the case of different broadcast point intervals, the location error of the three schemes becomes larger as the broadcast point interval becomes larger. In the case of the same broadcast position interval, the location errors of the three schemes are in descending order: PI scheme, DuRT scheme, and the scheme in this paper. This sorting indicates that the localization scheme proposed in this paper is far superior to the PI scheme and the DuRT scheme.

## 5. Conclusions and Future Work

In this paper, a novel mobile anchor node assisted RSSI localization scheme for UWSNs is proposed. After one mobile anchor travels along the planned linear trajectory on the water surface, all underwater sensor nodes in the area near the trajectory can locate their positions. We design an SVR-based interpolation method to improve the technique of determining node projection in existing methods. This method increases the accuracy of the nonlinear regression model of noisy measured data and decreases the estimation error caused by the discreteness of measured data, which greatly reduces the error of the determined projection and then improves the location accuracy of the underwater sensor node. By developing a curve matching method to obtain the perpendicular distance from the sensor nodes to the linear trajectory, the location of the sensor node can be calculated based on the determined projection and the obtained perpendicular distance. Compared with existing schemes that require the anchor node to travel at least two trajectories, the proposed scheme only needs one trajectory to locate sensor nodes, and the location time is shortened with the reduction in the number of trajectories. Simulation results show the validity of our proposed localization scheme, the SVR-based interpolation method, and the curve matching method. In the future, we aim to enhance the curve matching method to reduce the error between the obtained perpendicular distance and the actual perpendicular distance, so as to further improve the location accuracy.

## Figures and Tables

**Table 1:** Main notations used in this paper.

Notation	Definition
*L*	The length of the trajectory
ΔL	The interval between adjacent broadcast points
*m*	The number of broadcast points on the trajectory
$x_{i}$	The x-coordinate of the *i*th broadcast point
X	The location vector consisting of x-coordinates of all broadcast points
$r_{i}$	The noiseless RSSI value of the message from the *i*th broadcast point
R	The noiseless RSSI value vector
${\tilde{r}}_{i}$	The measured RSSI value of the message from the *i*th broadcast point
$\tilde{R}$	The measured RSSI value vector
${\hat{r}}_{i}$	The predicted RSSI value of the message from the *i*th broadcast point
$\hat{R}$	The predicted RSSI value vector
${\overset{`}{x}}_{ik}$	The x-coordinate of the *k*th point inserted after the *i*th broadcast point
$\overset{`}{X}$	The extended location vector
${\overset{`}{r}}_{ik}$	The RSSI value of the *k*th point inserted after the *i*th broadcast point
$\overset{`}{R}$	The extended RSSI value vector
$R_{i}$	The reference RSSI value vector of the *i*th reference trajectory
$d_{i}$	The perpendicular distance corresponding to the *i*th reference trajectory
⌊·⌋	Floor function
$\overset{ˇ}{X}$	The vector formed by the location of all feature points
$\overset{ˇ}{R}$	The vector formed by the RSSI value of all feature points
$\bar{X}$	The vector formed by the location of selected feature points
${\bar{R}}_{i}$	The vector formed by the RSSI value of selected feature points on the *i*th reference trajectory

**Table 2:** Parameters used in the simulation.

Parameter	Value	Parameter	Value
*L*	600 m	*H*	100 m
*N*	100	ΔL	1,5,10,15,20 m
$\Delta L_{ext}$	0.5 m	Δd	1 m
SL	100 db	*f*	24 KHz
*k*	2	*v*	5 m/s
$cd_{s}$	100 m	$cd_{a}$	600 m
